# Moyamoya syndrome associated with neurofibromatosis type I in a pediatric patient

**DOI:** 10.1590/S1516-31802011000200010

**Published:** 2011-03-03

**Authors:** Luiz Guilherme Darrigo, Elvis Terci Valera, André de Aboim Machado, Antonio Carlos dos Santos, Carlos Alberto Scrideli, Luiz Gonzaga Tone

**Affiliations:** I MD. Pediatric Oncologist, Division of Pediatric Oncology, Department of Pediatrics, Ribeirão Preto School of Medicine, University of São Paulo, Ribeirão Preto, São Paulo, Brazil; II MD, PhD. Attending Physician, Division of Pediatric Oncology, Department of Pediatrics, Ribeirão Preto School of Medicine, University of São Paulo, Ribeirão Preto, São Paulo, Brazil; III MD. Radiologist, Division of Diagnostic Imaging, Department of Internal Medicine, Ribeirão Preto School of Medicine, University of São Paulo, Ribeirão Preto, São Paulo, Brazil; IV MD, PhD. Full Professor, Division of Diagnostic Imaging, Department of Internal Medicine, Ribeirão Preto School of Medicine, University of São Paulo, Ribeirão Preto, São Paulo, Brazil; V MD, PhD. Full Professor, Division of Pediatric Oncology, Department of Pediatrics, School of Medicine of Ribeirão Preto, University of São Paulo, Ribeirão Preto, São Paulo, Brazil; VI VIMD, PhD. Titular Professor, Division of Pediatric Oncology, Department of Pediatrics, Ribeirão Preto School of Medicine, University of São Paulo, Ribeirão Preto, São Paulo, Brazil.

**Keywords:** Stroke, Magnetic resonance imaging, Moyamoya disease, Neurofibromatosis 1, Pediatrics, Acidente cerebral vascular, Imagem por ressonância magnética, Doença de moyamoya, Neurofibromatose 1, Pediatria

## Abstract

**CONTEXT:**

Neurofibromatosis type 1 (NF-1) is the most prevalent autosomal dominant genetic disorder among humans. Moyamoya disease is a cerebral vasculopathy that is only rarely observed in association with NF-1, particularly in the pediatric age range. The present study reports an occurrence of this association in an infant.

**CASE REPORT:**

An eight-month-old female presented convulsive seizures with clonic movements. The patient suffered an ischemic stroke with hemiparesis. Magnetic resonance imaging revealed radiological findings compatible with moyamoya disease. The diagnosis of NF-1 was made at the age of 20 months.

**CONCLUSION:**

Despite the rarity of this association in childhood, children with focal neurological symptoms and a diagnosis of NF-1 deserve to be investigated for moyamoya syndrome.

## Abstract (pt)

**CONTEXTO:**

Neurofibromatose tipo 1 (NF-1) é a doença genética autossômica dominante mais prevalente no ser humano. A doença de moyamoya é uma vasculopatia cerebral que raramente se encontra associada à NF-1, particularmente na faixa etária pediátrica. Este estudo descreve a ocorrência desta associação em um lactente.

**RELATO DE CASO:**

Paciente feminina, aos oito meses de idade, apresentou quadro de crise convulsiva com movimentos clônicos. Evoluiu com acidente vascular encefálico isquêmico e com hemiparesia à direita. Ressonância nuclear magnética mostrou achados compatíveis com a doença de moyamoya. O diagnóstico de NF-1 foi realizado aos 20 meses de vida.

**CONCLUSÃO:**

Apesar da raridade desta associação na faixa etária infantil, crianças com sintomas neurológicos focais e diagnóstico de NF-1 merecem ser investigadas para síndrome de moyamoya.

## INTRODUCTION

Neurofibromatosis type 1 (NF-1) is a multisystemic genetic disorder that displays important cutaneous manifestations such as *café-au-lait* spots, freckles and neurofibromas. Despite variable clinical expression, mutation of the *NF-1* gene is considered to be the most common *de novo* spontaneous autosomal dominant genetic alteration in human beings, with complete penetrance.^1^ The incidence of NF-1 is approximately one in 2,500 births, affecting all races. About 80,000 cases are estimated to exist currently in Brazil, and about 1.5 million worldwide.^1,2^

The *NF-1* gene is located on the long arm of chromosome 17, and more precisely, in the 17q11.2 band. This gene codes for neurofibromin, a protein that acts during nervous tissue growth remodeling.^2^ Recent studies have also demonstrated the presence of neurofibromin on the walls of vascular endothelial cells and in vascular smooth muscle cells.^3^

Moyamoya disease (MMD) is a rare inherited cerebral disorder of unknown etiology characterized by obliteration of the internal carotid artery and its branches, with the concomitant development of an abnormal network of collateral vessels. Moyamoya syndrome (MMS), an acquired form of MMD, also displays the angiographic pattern of MMD, although it is usually associated with different risk factors such as NF-1, Down syndrome and previous cranial irradiation, among others.^4^ Associations between NF-1 and vascular disorders are not uncommon, although associations between NF-1 and specific cerebrovascular disorders such as MMS are far less frequent.^5,6^

The objective of this study was to report on the case of a patient with NF-1 and MMS. This paper also provides a brief review of the main clinical and radiological aspects of this association. We conducted a systematic search in the PubMed, Cochrane Library, Lilacs (Literatura Latino Americana e do Caribe em Ciências da Saúde) and SciELO (Scientific Electronic Library Online) databases. The results obtained are shown inTable 1.

**Table 1.** Systematic review of the literature

Database	Search strategy	Results
PubMed	Neurofibromatosis type 1 [MeSH] AND moyamoya disease [MeSH]	28 articles
		23 case reports
		2 original articles
		2 reviews
		1 case series
Lilacs	Neurofibromatosis type 1 AND moyamoya disease	No articles
SciELO	Neurofibromatosis type 1 AND moyamoya disease	1 case report
Cochrane	Neurofibromatosis type 1 AND moyamoya disease	No articles

## CASE REPORT

**Figures 1A-1F.**
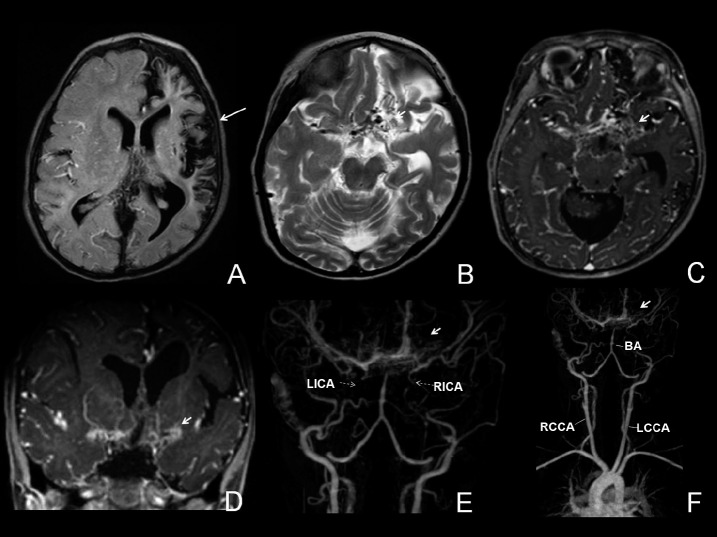
Magnetic resonance imaging performed with Philips 3.0 Tesla apparatus showing FLAIR (fluid-attenuated inversion recovery) images (A), T2 (B), post-contrast T1 (C and D) and angioresonance of intracranial and cervical vessels (E and F). Asymmetry of the brain hemisphere can be seen in A, with marked atrophy on the left, distortion of the gyri and prominence of the sulci, involving the cortex and the left frontotemporal-parietal white matter, while sparing the ipsilateral occipital region (large arrow). B, C and D show exuberant tortuous collateral circulation above the occlusion of the supraclinoid internal carotids. E and F show occlusion of the internal carotids in the supraclinoid region and above the basilar artery, associated with exuberant and tortuous collateral circulation in perforating vessels (dotted and small arrows).

LEGEND: LICA = left internal carotid artery; RICA = right internal carotid artery; BA = basilar artery; RCCA = right common carotid artery; LCCA = left common carotid artery.

An eight-month-old Afro-descendant girl was referred to the emergency room of the University Hospital due to convulsive 5–10 minute seizures characterized by mucosal pallor, clonic movement to the right and right-sided head version. The patient presented postictal somnolence. No fever was observed at the time of the seizures. A cerebrospinal fluid tap yielded normal results. Brain magnetic resonance imaging (MRI) revealed asymmetry of the hemispheres, with hemiatrophy on the left (Figure 1A) and signs of internal carotid occlusion in the supraclinoid and basilar portions, with marked collateral circulation through perforating vessels, with a moyamoya pattern (Figures 1B to 1F). Hemiparesis was most evident in the right upper limb.

A diagnosis of ischemic stroke was made based on the clinical and radiological findings. Thrombophilia testing was negative. At one year and eight months of age, she was referred to the medical genetics service due to the presence of skin lesions, delayed motor development and facial dysmorphism. Physical examination revealed several *café-au-lait* spots (larger than 0.5 cm) and cutaneous neurofibromas. At two years of age, the patient presented facial paralysis with slight facial asymmetry of peripheral pattern. Her mother also fulfilled the diagnostic criteria for NF-1. The girl is the first child of non-consanguineous healthy parents. Regarding her neuropsychomotor development, she was able to hold her head up at the age of seven months, spoke her first words at the age of one year and seven months and sat up unsupported at the age of one year and nine months. The child is currently under clinical surveillance, and persists with motor deficits.

## DISCUSSION

NF-1 occurs most frequently during childhood, and its diagnosis is based on the clinical criteria established by the National Institutes of Health (NIH) Consensus Development Conference.^2,7^ For a definitive diagnosis of NF-1, two or more of the following clinical characteristics must be present: I) six or more *café-au-lait* spots with a diameter of about 5 mm in prepubertal individuals, or with a diameter of more than 15 mm in postpubertal individuals; II) two or more neurofibromas of any type or one plexiform neurofibroma, based on clinical and histological parameters; III) freckles in an axillary or inguinal region; IV) optic glioma; V) two or more Lisch nodules (pigmented iris hamartomas); VI) a distinct bone lesion such as pseudarthrosis of a long bone or dysplasia of the sphenoid wing; and VII) a first-degree relative with NF-1 fulfilling the above criteria.

A variety of vascular lesions have been observed in patients with NF-1, such as arterial occlusion, aneurysms, pseudoaneurysms, stenosis, fistulae and ruptures. Although the arterial system is more commonly affected, lesions of the venous system have also been observed.^3^ The main characteristic of vascular lesions in patients with NF-1 is occlusion of the lumen and hyperplasia of the intima wall. Based on microscopic evaluation of the affected vessels, it has been proposed that the vasculopathy of NF-1 patients results from abnormal neurofibromin function that leads to excessive proliferation of vascular smooth muscle cells during normal maintenance of the vessel. Conversely, even though neurofibromin is known to be expressed in vascular smooth muscle cells, little is known about its function relating to controlling endothelial cell proliferation.^8^ Interestingly, despite the proximity of the *NF-1* gene (17q11.2) to the gene for familial moyamoya disease (17q25), NF-1 does not participate in occurrences of moyamoya disease.^9^

MMD was first described in 1957 and, since this initial report, much has been described regarding the clinical characteristics of the disease. Nonetheless, its etiology continues to be ill-defined.^10^ MMD is more frequently observed in the Japanese population, with an estimated incidence of one new case per 1,000,000 individuals per year. MMD more frequently affects children younger than 10 years.^11^

In children, cerebral ischemia has been the most common presentation of MMD. In a study on 143 pediatric patients with MMD in North America, Scott et al.^12^ observed that nearly all the patients had clinical symptoms of aneurysm or transitory ischemic attacks, and similar results have been reported in European studies.^13^ In a cohort study on 316 children with NF-1, Rosser et al.^3^ observed cerebral vasculopathy in eight of them (2.5%), with only two cases (0.6%) also presenting MMS.

Most patients with NF-1 associated with vascular lesions are asymptomatic.^14^ When symptoms are present, they include neurological findings such as paresthesia, headache, epileptic seizures, hemianopsia, nystagmus, aphasia, dysphasia and borderline mental level.^15^ Surgical intervention has become the treatment of choice for patients with MMD, and particularly surgical revascularization in order to increase the blood flow to the hypoperfused cortex.^5,16^

## CONCLUSION

The present report describes an additional case of associated NF-1 and MMS in a pediatric patient. Although this association is relatively uncommon, MMS is a potentially severe disease that may evolve with an unfavorable neurological course. Thus, the hypothesis that this association may be present should be considered in cases of patients with NF-1 and focal neurological symptoms, so that proper care can be promptly provided.

## ACKNOWLEDGMENTS

The authors would like to thank Dr. Maria Sol A. Brassesco for assistance in the literature review
